# Impact of the Nano-Precipitation Size Distribution on the Mechanical Behavior of Nickel-Based Alloys by Experiment and Simulation

**DOI:** 10.3390/nano15231759

**Published:** 2025-11-24

**Authors:** Yuebing Wen, Yunlong Liu, Shuhua Teng, Ruixue Yuan, Yuwei Song, Shiyuan Sun, Song Cai, Zhou Li, Bowen Liu, Dan Gao, Yang Chen

**Affiliations:** 1 College of Intelligent Manufacturing, Hunan First Normal University, Changsha 410205, China; ybwen@hnfnu.edu.cn (Y.W.);; 2 State Grid Hunan Electric Power Company Research Institute, Changsha 410007, China; 3 College of Electronic Information, Hunan First Normal University, Changsha 410205, China; 4 SEG Automotive Products (China) Co., Ltd., Changsha 410205, China; 5 State Key Laboratory of Advanced Design and Manufacturing Technology for Vehicle, College of Mechanical and Vehicle Engineering, Hunan University, Changsha 410082, China

**Keywords:** nickel-based superalloys, size distribution, mechanical behavior, dislocation dynamics simulation, machine learning

## Abstract

The outstanding mechanical properties of nickel-based alloys are predominantly governed by the characteristics of their nano-precipitation. Traditional optimization approaches, however, have focused almost exclusively on average precipitation size, neglecting the non-uniform size distribution induced by the temperature gradient during the actual preparation process. This oversight leads to inaccurate optimization parameters, hindering the reliable design and broader engineering application of these alloys. This study investigates the coupling effect of average size and deviation on the mechanical behavior of nickel-based superalloys by experiment and simulation. The characterization of prepared nickel-based alloys confirms the spatial non-uniform distribution of the precipitation size and calibrates dislocation dynamics (DD) simulation inputs. When the precipitation size exhibits no deviation, conventional strengthening models align with simulation results, confirming the accuracy of the simulations. Under high size deviation, however, significant discrepancies arise between traditional models and simulation outcomes, indicating that conventional models fail to account for the effect of size deviation. This is because size deviation leads to spatial heterogeneity in precipitate distribution, which is difficult to describe using a unified theoretical framework. Machine learning (ML)-augmented analysis of the extensive DD dataset reveals a previously unrecognized synergistic effect governed by precipitation size heterogeneity. Surprisingly, contrary to the conventional preference for a uniform size distribution, we find that an appropriate size deviation increases the number of precipitates that effectively pin dislocations in three-dimensional space, thereby enhancing strength at a constant volume fraction. Our combined experimentally calibrated DD/ML approach identifies an optimal range of size variation that maximizes strengthening potential. These results provide a new strategy and an effective pathway for performance optimization of nickel-based alloys.

## 1. Introduction

Nickel-based superalloys that are strengthened through nano-precipitation hardening possess outstanding strength, superior ductility, and lasting microstructural stability at elevated temperatures [1,2]. The exceptional durability of these alloys is mainly attributed to the presence of coherent γ’ precipitations within a face-centered cubic γ matrix [3,4]. Utilizing precipitation strengthening has become a prevalent method to enhancing the yield strength of metal-based materials [5,6,7,8]. These precipitations impede dislocation motion, thereby elevating the critical resolved shear stress (CRSS) [9]. The impact of geometrical factors, such as the size, shape, and volume fraction of precipitations on their strengthening effect, has been extensively examined [10,11]. However, previous studies have primarily concentrated on average spacing and size, neglecting the crucial influence of the random spatial distribution of precipitations on their strengthening contribution.

The critical precipitate diameter marks the point where dislocation pair interactions shift from weak to strong coupling, coinciding with maximum strength [12,13]. The size distribution of precipitations is influenced by aging and cooling rates in Ni-based alloys. The coarsening of precipitations requires elevated temperatures due to a balance between interfacial and elastic energies [14]. Optimal performance is achieved via carefully designed heat treatment schedules, since precipitate morphology and dimensions critically govern precipitation hardening effectiveness.

Recent research [15] has underscored the importance of the random spatial distribution of obstacles in strengthening behavior. Nevertheless, these studies often overlook the impact of precipitation size and its deviation, thus limiting our comprehension of randomly distributed precipitations. Specifically, the small gap between precipitation size and spacing in nickel-based superalloys leads to a non-negligible size deviation [16]. Molecular dynamics (MD) and discrete dislocation dynamics (DDD) simulations elucidate precipitation strengthening mechanisms by probing dislocation interactions with precipitate ensembles [17,18,19]. MD simulations offer detailed insights into microstructural evolution at the atomic level [18], while DDD simulations observe dislocation–collective precipitation interactions on larger spatial and temporal scales [20]. Experimental evidence indicates that precipitations exhibit a non-uniform spatial distribution with size deviations, rather than uniform arrays [21]. This results in enriched precipitation size and spatial distribution, significantly affecting mechanical properties [21]. Further investigation is required to enhance precipitation strengthening by examining the impact of precipitation size dispersity and stochastic arrangement on CRSS.

MD calculates the motion of individual atoms directly through interatomic potentials, enabling precise revelation of dislocation core structures and fundamental interaction mechanisms [18]. However, its prohibitive computational cost restricts simulations to the nanoscale and nanosecond timescales, making it difficult to study macroscopic plastic behavior. In contrast, DDD treats dislocations as continuous elastic defects and simulates their evolution based on line tension theory and elasticity theory, thereby bridging micro to millimeter scales and longer realistic timeframes while significantly improving computational efficiency. This advantage comes at the expense of atomic-scale details and requires key parameters to be predetermined through molecular dynamics or experiments. The particular strength of DDD lies in investigating precipitate–dislocation interactions: it efficiently simulates the collective evolution of numerous dislocations in complex environments containing hundreds or thousands of precipitates-capturing mechanisms such as Orowan looping and shearing, which directly computes resulting work-hardening curves and critical shear stresses [20]. This enables quantitative establishment of the relationship between precipitate characteristics and macroscopic strengthening effects at the mesoscale.

Therefore, the aim of this paper is to enhance our comprehension of precipitation size enrichment and spatial distribution in strengthening behavior. By employing DDD simulations, we thoroughly investigate the interactions between precipitations with size deviations and dislocations in a three-dimensional context, elucidating the mechanisms of size enrichment and the strengthening effect of spatial distribution. Furthermore, machine learning is utilized to ascertain the optimal size deviation and average size effect.

## 2. Experiment

### 2.1. Material Preparation

The nickel-based superalloy investigated in this work has a nominal composition (in wt.%) of Ni (balance), Co 26.0, Cr 13.0, Mo 4.0, W 4.0, Al 3.2, Ti 3.7, Nb 0.95, C 0.05, B 0.025, Zr 0.05, and Hf 0.2. Production involved vacuum induction melting followed by argon gas atomization, yielding spherical powder (50–150 μm diameter). The powder was housed in a stainless-steel container, vacuum degassed (400 °C, 24 h), and sealed. Subsequent processing included hot isostatic pressing (HIP: 1100 °C, 140 MPa, 4 h). After container removal, the HIPed billet underwent hot extrusion (HEX) following a 2 h soak at 1100 °C, achieving a 6:1 area reduction ratio.

### 2.2. End Quenching Test

A Jominy specimen, machined from the as-HEX billet to dimensions of 25 mm diameter and 100 mm length, was solution-treated at 1180 °C for 40 min (exceeding the approximate solvus temperature of 1154 °C). The sample, coated with ceramic fiber wool and exposing only the bottom end for gas quenching, was immediately quenched. Cooling rates (120 °C/min) throughout the probe were simulated using specialized software (MATLAB 2022) implementing a one-dimensional unsteady heat-conduction differential equation. Physical parameters, determined via laser flash apparatus (FLA, Netzsch LFA 457 MicroFlash, Selb, Germany) experiments, were calibrated against thermocouple measurements taken at three points along its length.

### 2.3. Microstructure Characterization

Field emission scanning electron microscopy was employed to investigate the spatial distribution of precipitations (Quanta 650 FEG, FEI, Hillsboro, OR, USA) at 20 kV in secondary electron imaging mode, alongside transmission electron microscopy (Titan G2 60–300, FEI) functioning at 300 kV. ImageJ software [22] was employed to ascertain the size and spacing of γ’ precipitations from SEM images.

## 3. Methods

### 3.1. Dislocation Dynamics Simulation

Every DDD simulation in our work leverages the software ParaDiS (for the specific user manual of ParaDis software), initially crafted at Lawrence Livermore National Laboratory [17]. The parameters for nickel-based DDD simulations are outlined in Table 1. Notably, consistent precipitation volume fractions and mean radii were observed throughout the DDD simulations. These utilized a 2 μm cube simulation box featuring full periodic boundary conditions to model bulk crystal behavior [23]. An edge dislocation was introduced on a designated plane. This study utilized a strain rate of 1 × 10^3^ s^−1^, in line with previous DDD simulations [24]. The precipitation radius follows a log-normal distribution, consistent with experimental observations [25]. Its probability density function (PDF) is derived from [26]:

(1) $$f_{1}(r)=\frac{1}{\sqrt{2\pi }r\phi }exp\left[-\frac{(ln(r)-\xi {)}^{2}}{2\phi^{2}}\right]$$

The representative geometric value:

(2) $$\xi =ln\left(\frac{E^{2}}{\sqrt{D+E^{2}}}\right)$$

Based on experimental measurements of precipitation particle size, where *E* denotes the arithmetic mean size and *D* represents the arithmetic size variance, the geometric standard deviation (*ϕ*) is expressed as

(3) $$\phi =\sqrt{ln\left(\frac{D}{E^{2}}+1\right)}$$

**Figure 1:**
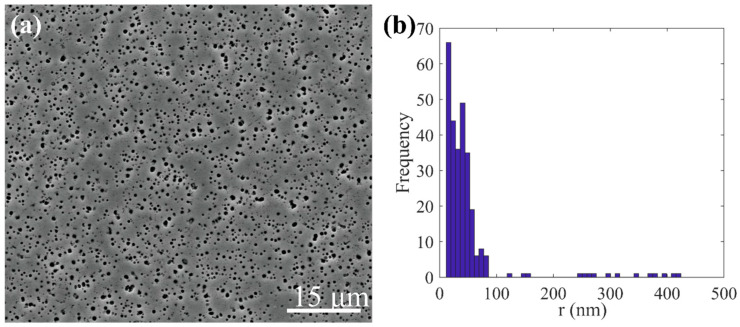
(**a**) SEM image showing the space distribution of precipitation. (**b**) Statistical analysis of precipitate radial dimensions.

Experimental analysis indicates a volume fraction of 0.3 (Figure 1a), with a mean and standard deviation for precipitation radius of around 50 nm and 64 nm (Figure 1b). ImageJ software was used to calculate the volume fraction of the precipitated phase in the experimentally obtained images. To mirror actual conditions, the parameters for precipitation distribution used in the DDD simulations fluctuated within experimentally determined values, detailed in Table 1. These parameters were integrated into DDD simulations to evaluate how precipitation size non-uniformity dictates the mechanical responses of nickel-based superalloys. The relationship between the average radius of precipitations and the center-to-center spacing *L* is described by the equation $f_{\nu }=\frac{2\pi }{3}{\left(\frac{\bar{r}}{L}\right)}^{2}$, where $f_{\nu }$ represents the proportion of the total volume occupied by the precipitate.

**Figure 2:**
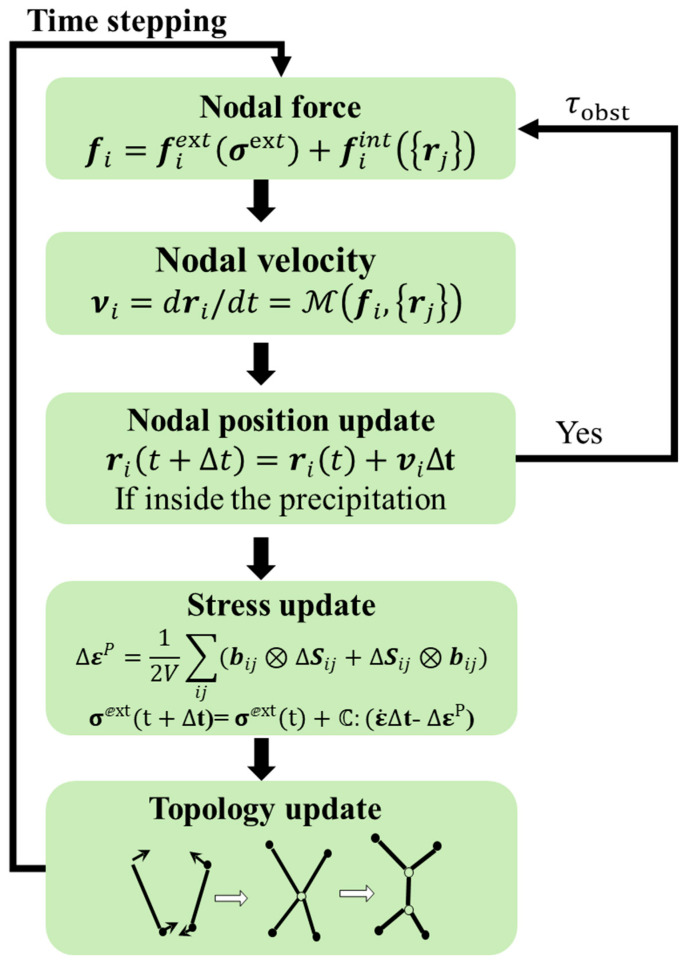
Flowchart of the DDD calculation.

The strengthening influence of precipitations hinges on their properties, such as coherency and order [27]. An antiphase boundary creates a resisting force that prevents dislocations from cutting through precipitations in nickel-based superalloys [20]. Incorporating elastic modulus mismatch and modeling precipitates as hardening agents yields a higher CRSS, while established theory [27] shows coherent strengthening contributes only secondarily to the CRSS increment. The calculation results in Appendix A also match this conclusion. To address computational load, a simplified model is chosen to accurately examine the effects of size variance on the precipitation strengthening mechanism. Consequently, weak stacking-fault and coherency effects are disregarded. Therefore, the interaction between dislocations and precipitations is modeled as described by [28,29,30]:

(4) $$\tau_{obst}=\left\{\begin{array}{c} -\gamma_{APB}/b\;\;\;\;\;\;\;\;\;\;inside\;a\;particle\\ 0\;\;\;\;\;\;\;\;\;\;\;\;\;\;\;\;\;\;\;\;\;\;\;\;\;\;\;\;\;\mathrm{outside} \end{array}\right.$$

where $\gamma_{APB}$ signifies anti-phase boundary energy, while b denotes the magnitude of Burgers vector. The process of the DDD calculation is shown in Figure 2.

**Table 1:** The material parameters of Ni-based superalloys for DDD simulation parameters.

Parameter	Symbol	Value
Magnitude of the Burgers vector	*b*	0.248 nm [31]
Dislocation core size	r_0_	1 *b*
Shear modulus	μ	80 GPa [31]
Poisson’s ratio	v	0.31
Drag coefficient	B	2.5 × 10^−4^ (Pa·s) [32]
Anti-phase boundary energy	$\gamma_{APB}$	0.27 J/m^2^ [33]
Precipitation mean size	d	161.4 nm [34]
Volume fraction	*f_v_*	0.245 [34]

### 3.2. Machine Learning Method

**Figure 3:**
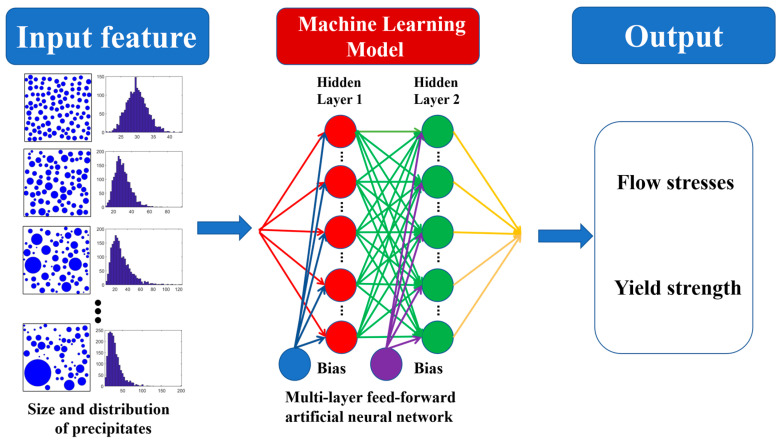
The architecture of ANN for searching for the optimum range of precipitation sizes and distributions in nickel-based alloys with high yield strength/flow stresses.

Machine learning is used to search for the optimum ranges of precipitation size and distribution in nickel-based alloys with high yield strength/flow stresses, as shown in Figure 3. Machine learning usually has five main steps [35,36], as follows: (i) Collect the dataset. The dataset includes the yield strength and flow stresses with different precipitation sizes and distributions. These data points come from DDD simulations. It is worth noting that the three-dimensional radii of these precipitates are calculated, and the resulting size distribution is input into a machine learning model. The parameters of the precipitated phase come from two approaches. One approach is to calculate the cumulative area of the precipitated phase pixels in the experimental images and then obtain the radius through the formula for the area of a circle. The other one is to directly derive the parameters of the simulated precipitated phases from the discrete radius data of each precipitated phase given during the DDD simulation modeling. The average size of the precipitated phases in the experiment and DDD simulation was obtained by averaging all the precipitated phases. The distribution of the precipitated phase is quantified by calculating the variance of the radii of the discrete precipitated phase. The yield strength and flow stress are derived from the stress–strain curves of different precipitated phase sizes and variances simulated by DDD simulation. Among them, the yield strength is taken from the first drop point of the stress–strain curve, while the flow stress is derived from the average stress after the first drop point of the stress–strain curve. To effectively train the machine learning model, the input and output features have been normalized, which can be described as $X^{\prime }=\frac{X-Min(X)}{\mathrm{Max}\left(X\right)-Min(X)}$. The full list of the features used for machine learning is presented in Table 2. (ii) Train the machine learning model. Here, the original dataset, including data from 49 DDD simulations (Appendix B, Table A1), is divided into three parts randomly. The training set (60%) estimates the parameters, the tuning (validation) set (20%) tunes the hyper-parameters, and the assessment (test) set (20%) independently assesses the performance of the artificial neural network (ANN) model. The multi-layer feed-forward ANN is used to create the relationship between precipitation features and mechanical properties (Figure 3). The multi-layer ANN is selected because the relationship between precipitation features and mechanical properties is highly complex and nonlinear. While fewer features were used, their interaction with properties is still intricate. Traditional linear or simpler models cannot adequately capture this complexity. Tensorflow, an end-to-end open-source machine learning platform, is used to construct the ANN model [37]. (iii) Use t coefficient to diagnose predictive fidelity through observed-versus-estimated harmonization. (iv) Export the best machine model. (v) Employ the best machine model to predict the yield strength/flow stresses with different precipitation features.

An ANN is a multi-layered system where data flow from input to output and shaped and transformed by hidden layers packed with interconnected nodes [37] (Figure 3). The input layer is applied to receive the input features (precipitation features). The hidden layers carry out the calculations, which extract and process the feature information from the input layer. The output layer can give the prediction based on the results from the hidden layers. It should be noted that all layers are forward-connected. In ANN, all neurons can handle and transmit the information. Specifically, the output of *j*th neuron, $a_{j}$, is written as $a_{j}=\sum_{i=1}^{n}\omega_{i}\cdot x_{i}+b_{j}$, where $\omega_{i}$ represent the weights connected with the *i*th input neuron $x_{i}$, and $b_{j}$ denotes the bias term. In addition, the sigmoid function, $Sig\left(x\right)=\frac{1}{1+e^{-x}}$, is used as activation function, and the Levenberg–Marquard algorithm is chosen as optimizer for optimizing the $\omega_{i}$ and $b_{j}$ based on the error between the target output and predicted output [38].

### 3.3. Classical Precipitation-Strengthening Model

**Figure 4:**
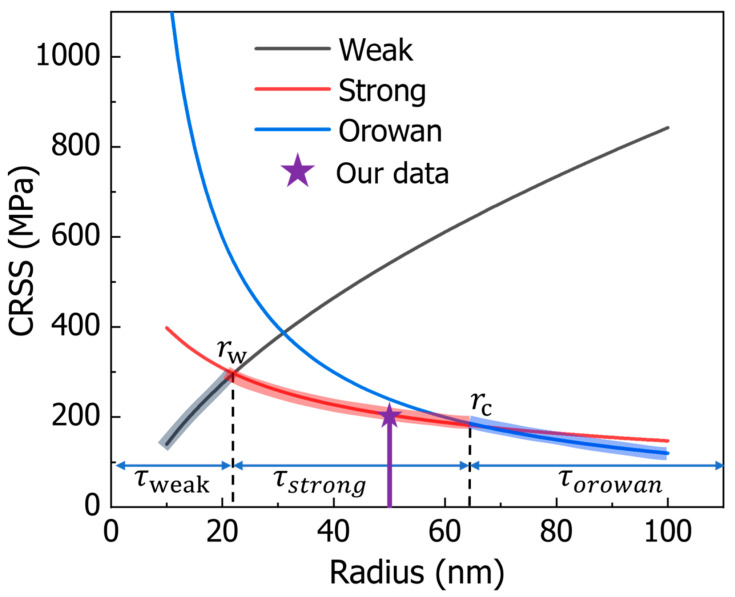
The relationship between strengthening effects and precipitation size can be characterized as follows: $r_{w}$ serves as the critical dimension that marks the shift from weak-pair to strong-pair coupling modes, while $r_{c}$ acts as the critical size indicating the transition between the cutting and looping mechanisms. Here, our data are for the average size of the precipitation in the nickel-based alloy prepared in our experiment.

The classic precipitation strengthening mechanism is closely related to the average radius of the precipitation, and there are three types of strengthening mechanisms corresponding to the precipitation with radii ranging from small to large. Two distinct scenarios emerge when comparing the diameter of precipitates to the distance between these paired dislocations. These are referred to as weak-pair coupling and strong-pair coupling, as illustrated in Figure 4. Weak-pair coupling occurs when the spacing between the two paired dislocations exceeds the precipitate diameter; in this instance, faulted precipitates are present in the region separating the two dislocations [28].

When a precipitate’s radius is smaller than $r_{w}$ (Figure 4), the CRSS for weak-pair coupling can be expressed as follows [31]:

(5) $$\tau_{weak}=\frac{\gamma_{APB}}{2b}\left[{\left(\frac{6\gamma_{APB}fr}{2\pi T}\right)}^{1/2}-f\right]$$

Here $\gamma_{APB}$ refers to the anti-phase boundary energy, b is the Burgers vector magnitude, f denotes the precipitate volume fraction, r is the precipitate radius, T (equal to Gb^2^/2) represents the dislocation line tension, and G stands for the shear modulus. When precipitates are large enough, (with sizes on par with the spacing between paired dislocations), the scenario shifts to strong-pair coupling, and it is possible for two paired dislocations to enter the precipitate at the same time.

For precipitate radii in the range between $r_{w}$ and $r_{c}$ (Figure 4), the CRSS under strong-pair coupling is given by [31]:

(6) $$\tau_{strong}=\sqrt{\frac{3}{2}}\left(\frac{Gb}{r}\right)\frac{f^{1/2}}{\pi^{3/2}}{\left(\frac{2\pi \gamma_{APB}r}{Gb^{2}}-1\right)}^{1/2}$$

The maximum CRSS value emerges when the weak-pair coupling model and the strong-pair coupling model meet, meaning when $\tau_{weak}=\tau_{strong}$ (Figure 4).

For precipitates with a radius larger than the critical radius $r_{c}$ (Figure 4), the CRSS required for dislocations to cut through the precipitate becomes greater than that needed for dislocations to go around the precipitate and form an Orowan loop. In such cases, the Orowan bypass mechanism comes into play, and the CRSS for this mechanism is as follows [39]:

(7) $$\tau_{orowan}=\frac{3Gb}{2L}$$

Here, L is the average spacing between precipitates, calculated as $L=(\frac{2\pi }{3f}{)}^{1/2}r$. Additionally, the transition from the strong-pair coupling mechanism to the Orowan bypass mechanism takes place when $\tau_{strong}=\tau_{orowan}$ (Figure 4). In this paper, the average size of the precipitated phase is 50 nm. On this basis, we adopted the traditional $\tau_{strong}$ calculation model to obtain the CRSS value.

**Figure 5:**
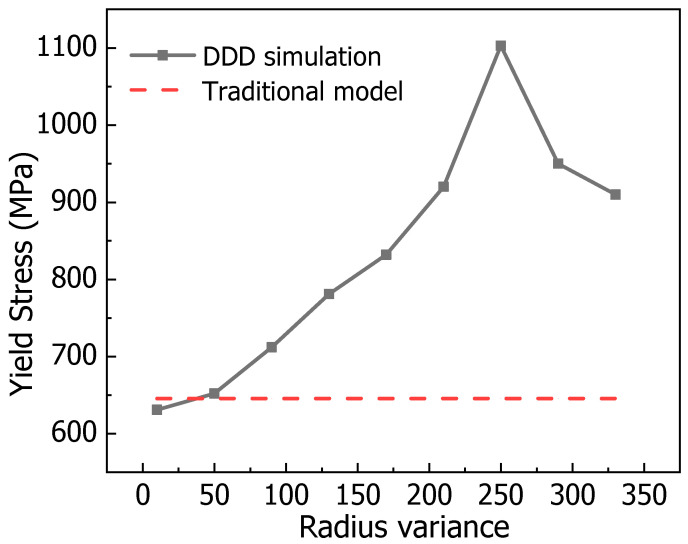
The yield stress without dimensional deviation obtained by the classical theoretical model is compared with the yield stress with dimensional deviation calculated by the simulation. The average size of the precipitated phase here is 50 nm.

For a nickel-based alloy with an average precipitation size of 50 nm, the yield strengths computed via DDD simulations under different size deviations are compared with values from the theoretical model (the model is verified through experiments) in Figure 5. The DDD results agree well with theoretical values at small deviations, confirming the accuracy of the simulations. As the deviation increases, however, the theoretical model increasingly overestimates the yield strength, revealing its inability to account for the influence of size dispersion. The spatial distribution of size deviations further complicates a unified theoretical description. Thus, we employ machine learning to construct a relationship between yield strength and the coupled effect of average size and size variance based on discrete simulation data.

## 4. Results and Discussion

### 4.1. Machine Learning Prediction

**Figure 6:**
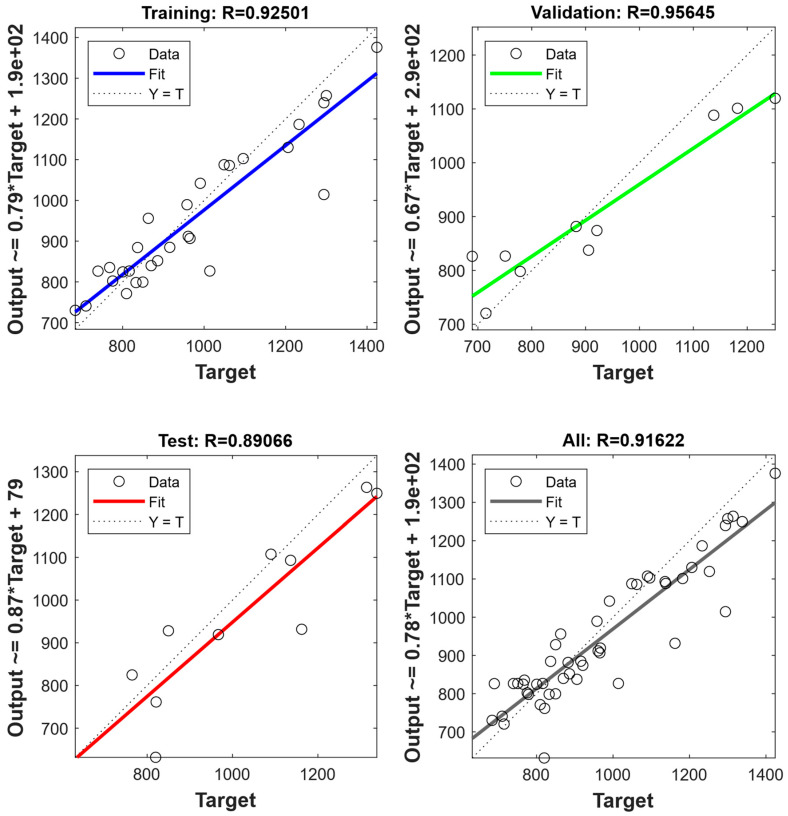
The performance of ANN model for yield strength.

**Figure 7:**
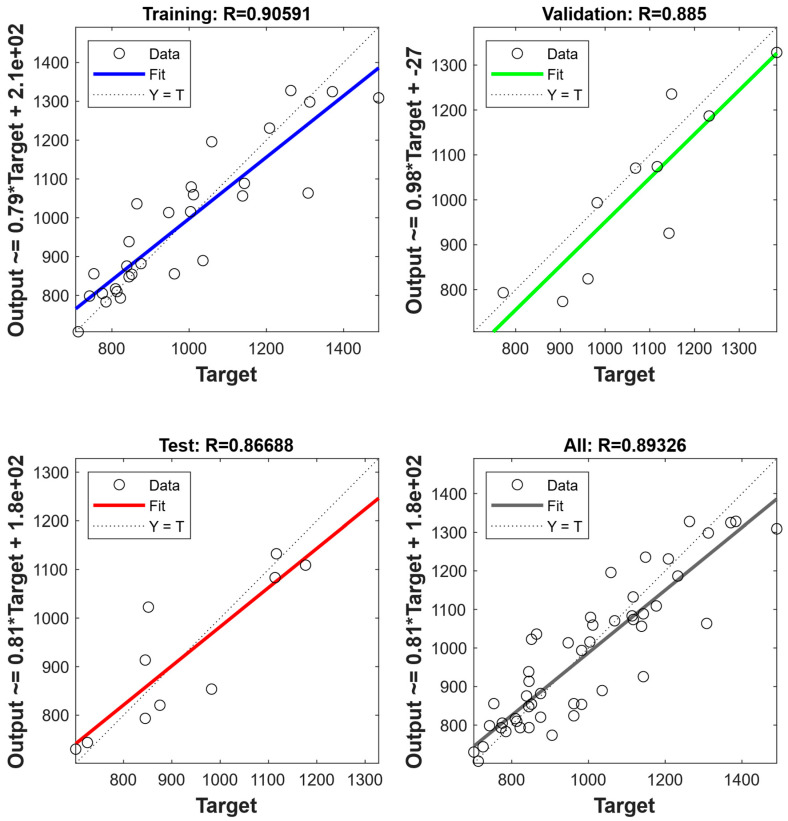
The performance of ANN model for flow stresses.

Figure 6 and Figure 7 illustrate the predicted values and target values of yield strength and flow stresses, respectively. The dotted line suggests a high degree of consistency between the predicted and target values. It can be found that in both the yield strength ANN model and flow stress ANN model, the data points in a learning set, a tuning set, and an assessment set, and the entire dataset all lie along the dotted line [40]. Moreover, the solid line represents a linear fit of the predicted values and target values. By evaluating the relationship between predicted values and target values, it is found that the prediction results are distributed both above and below the dotted line, with no apparent systematic underestimation. Further, the slope and coefficient of determination (R) of the linear fit are calculated. The results showed that the slopes of the fitted line are 0.916 and 0.893 for yield strength and flow stresses, respectively. This indicates that the predictions from ANN models exhibit good consistency and linear correlation with the actual values. The consistency of the solid and dashed lines indicates the performance of the ANN model [41]. The results suggest that the trained ANN yield strength model and flow stress model both have excellent performance and robustness.

**Figure 8:**
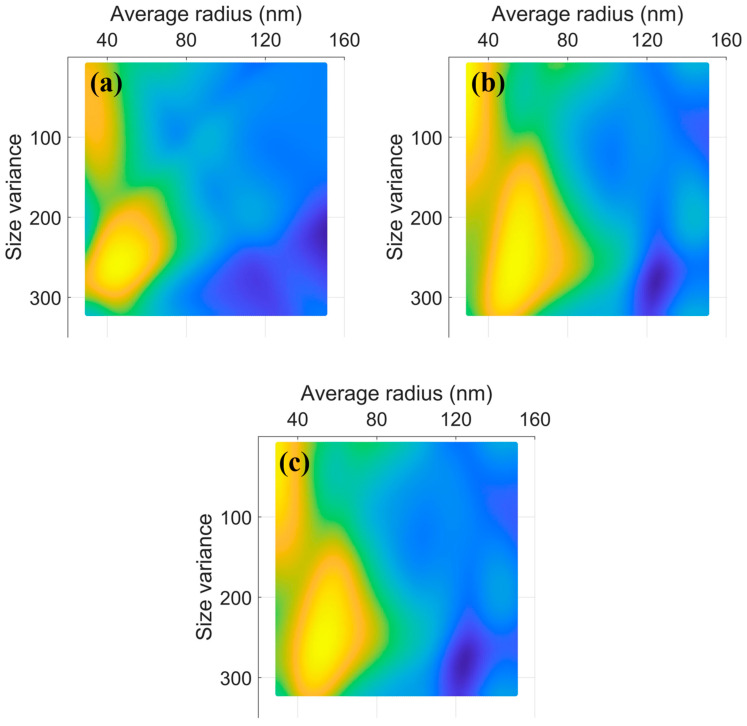
The distribution of yield strength (**a**), flow stresses, (**b**) and comprehensive property (**c**) with different precipitation sizes and distributions.

Based on the trained ANN model, the mechanical response characteristics of nickel-based alloy systems, including onset of plastic deformation and sustained stress under strain, with different precipitation sizes and distributions can be predicted instead of using DDD simulations. This method can traverse all possible cases of precipitation size and distribution in a few seconds. Figure 8a,b exhibits the distribution of the yield strength and flow stresses with precipitations of average size (from 30 to 150 nm) and size dispersion (from 10 to 320), respectively. Moreover, the parameter (Property=0.8×flow stresses+0.2×yield strength) is used to evaluate the comprehensive mechanical property. Figure 8c shows the distribution of the comprehensive mechanical property. The optimal values of the prediction are an average size between 40 and 70 nm and size dispersion between 160 and 300 (Figure 8c). By training a well-performing ANN model, an initial reference range for experiments can be provided.

### 4.2. Dislocation Evolution

**Figure 9:**
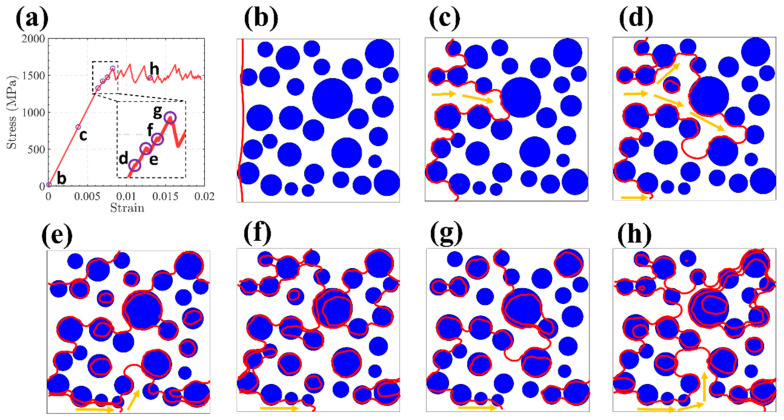
(**a**) The stress–strain curve for the sample at the average precipitation radius of 50 nm and the size variance of 240. (**b**–**h**) The dislocation configuration (red line 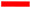) at different strains in (**a**). The blue ball 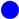 is the precipitation. The orange arrows (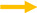) represent the free motion path of dislocation.

The spatial arrangement of precipitates within the alloy exhibits stochastic behavior, deviating significantly from uniform or periodic configurations. Therefore, discrete precipitate entities tend to spatially coalesce, leading to the development of chemically enriched microstructural domains [29], which become regions that are difficult for dislocations to cross. Therefore, it is necessary to study the path of dislocation over precipitation in detail to reveal the influence of spatial dispersion distribution of precipitation on CRSS. Based on the optimal precipitation parameters obtained by machine learning in the previous chapter (the average precipitation radius of 50 nm and the size variance of 240), we obtained the tensile stress–strain curve of the sample under this parameter by dislocation dynamics simulation (Figure 9a). Figure 9b–h show the spatial configuration of the dislocation under different tensile strains. The interaction between dislocations and precipitates was simulated using the ParaDis software. The spatial configurations of dislocations at varying strain levels were extracted from the simulations. These data, comprising nodal coordinates and connectivity matrices, were subsequently processed with MATLAB to generate the graphical representations. At the beginning of stretching, the dislocation is flat (Figure 9b). As the stress increases, the dislocation begins to bend, and the region with the largest precipitation spacing is selected to advance (Figure 9c). There are two rather wide regions in the precipitation spacing at the same time, and the dislocation will bend towards both regions at the same time (Figure 9d). In the agglomerated zone, the intervals between precipitates are narrower than the overall average, whereas in surrounding areas, the distances exceed this average (as depicted in Figure 9c,d). Consequently, dislocations preferentially traverse regions within the agglomerated zone where precipitate spacing is relatively wide (Figure 9d). This leads to the emergence of localized dislocation structures—resembling Orowan loops—encircling the region of precipitation-induced segregation (Figure 9e,f) [29,42]. These precipitation-segregation bands cause the dislocation slip path to be severely bent, resulting in higher dislocation line tension (Figure 9f,g) [43,44]. As the strain continues to increase, the formed dislocation “Orowan Island” does not increase, because the already-formed dislocation “Orowan island” shrinks inward to cut the precipitation under the extrusion of the new dislocation “Orowan Island” and finally disappears. Repeatedly, the dislocation configuration eventually reaches a state of dynamic equilibrium, resulting in fluctuating flow stresses (Figure 9a,h).

### 4.3. Average Size Effect

**Figure 10:**
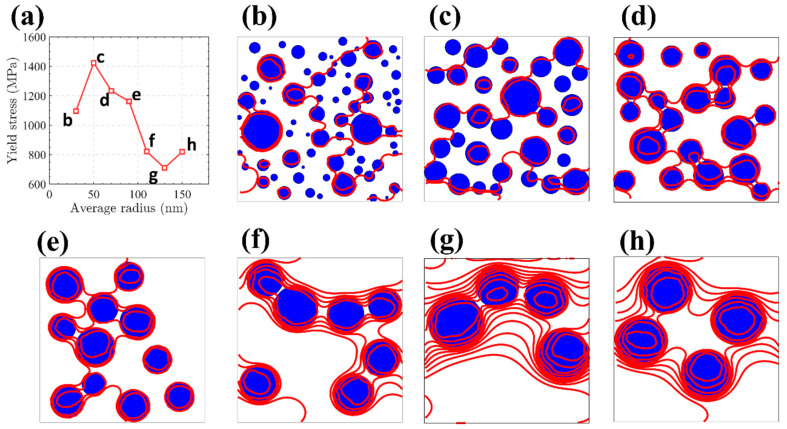
(**a**) Depicts how yield stress responds to changes in mean precipitate dimensions when the size variance is 240. (**b**–**h**) Present the corresponding dislocation patterns highlighted in red (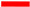) at various average precipitation sizes under the same size variance. These configurations represent dislocation lines spanning vertically across the simulation domain once the applied stress attains the CRSS threshold. The blue ball 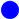 is the precipitation.

The average size of precipitation is one of the important characteristics of microstructure in nickel-based alloys. Therefore, a detailed analysis of how the mean precipitation size influences the mechanical behavior is crucial for refining the microstructural design of nickel-based alloys. Based on the optimal precipitation radius of 50 nm and the size variance of 240 obtained by machine learning, we explored dislocation evaluation for the precipitation with the size variance of 240 and the average radius of 50 nm. Figure 10a illustrates how the critical resolved shear stress (CRSS) varies in response to changes in the average radius of the precipitates. With the increase in precipitation mean size, CRSS increased first and then decreased. The change trend is very steep and obvious, indicating that there is indeed an optimal precipitation average radius to enhance the mechanical performance of nickel-based alloys by optimizing key microstructural parameters to reach peak efficiency. Figure 10b–h shows that the characteristic arrangement of dislocations subjected to critical resolved shear stress varies according to the mean precipitation size.

It is well known that under the same volume fraction and size variance of precipitation, the number of precipitations per unit area depends on its average size. When the average size increases, the number of precipitations will decrease, and the spacing between precipitations will increase. This trend will make it easier for dislocations to pass through the precipitation, resulting in a decrease in the hindrance of the precipitation’s relative dislocation [45]. On the other hand, as the number of dislocations decreases, the degree of curvature of the critical dislocation configuration also decreases (Figure 10b–h). This shortening of the dislocation pathway diminishes the associated energy and results in a lowered tension along the dislocation line [20]. This reflects that the CRSS forming the critical dislocation configuration decreases with the increase in precipitation mean size. However, the precipitation mean size is not the smaller the better. Because, when the average size of the precipitation is too low, the dislocation will directly cut the very small size of the precipitation. This shows that the hindering ability of precipitation relative to dislocations of very small sizes (less than 10 nm) is negligible. Many very small precipitations reduce the obstruction of the dislocation, resulting in a decrease in CRSS. In short, there is a suitable precipitation average size, so that the strength of the nickel-based alloy attains the highest value.

### 4.4. Size Dispersion Effect

**Figure 11:**
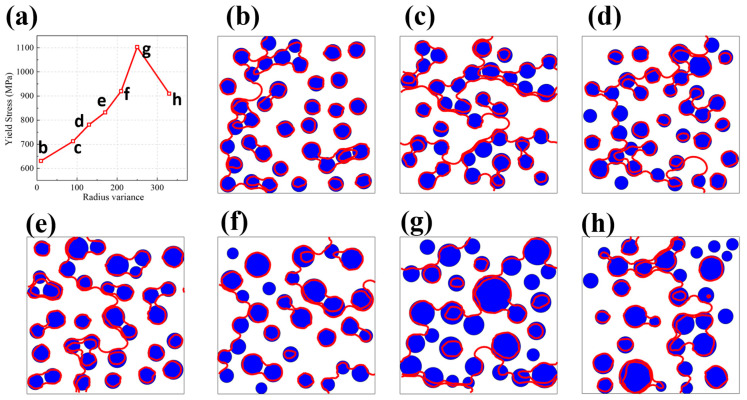
(**a**) Displays the correlation between yield stress and precipitation size variability, with the mean precipitate radius set at 50 nm. (**b**–**h**) Illustrate the distinctive dislocation arrangements marked in red (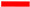), formed under CRSS conditions across varying degrees of precipitation size variance, all within the context of a consistent 50 nm average radius. The blue ball 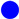 is the precipitation.

Since the size of the precipitation is not the same in every one, the size dispersion is one of the important characteristics of the microstructure in nickel-based alloys. Therefore, a comprehensive evaluation of how variations in precipitation size distribution impact mechanical characteristics is key to fine-tuning the microstructural configuration of nickel-based alloys. Using the optimal precipitation parameters (the average precipitation radius of 50 nm and the size variance of 240) obtained by machine learning, we investigated the influence of size variance on CRSS at the average precipitation radius of 50 nm. Figure 11a illustrates how variations in precipitation size distribution influence CRSS. As the dispersion widens, the CRSS initially rises before subsequently declining. The variation trend is very steep and obvious, indicating that there is indeed an optimal precipitation size dispersion to achieve the optimal mechanical properties of nickel-based alloys. Figure 11b–h show the critical dislocation configuration under CRSS at different size dispersions.

When the average precipitation size remains constant, an elevated dispersion in precipitation dimensions raises the likelihood of encountering smaller cross-sections along dislocation slip planes (Figure 11b–h). This phenomenon increases the total precipitation count per unit volume at fixed volume fraction, as finer precipitations achieve higher numerical density. Consequently, dislocations experience more frequent interactions with these obstacles (Figure 11g), inducing pronounced curvature in dislocation lines, which is a well-documented mechanism for strengthening [15,29]. The extended dislocation path necessitates greater energy expenditure during slip, thereby elevating the critical resolved shear stress (CRSS) [20]. However, extreme size variation produces a bimodal distribution of precipitation sections on slip planes. Oversized precipitations reduce obstacle density through sparse distribution, while subcritical particles below a threshold size [29] offer negligible resistance. This dual effect diminishes effective dislocation–precipitation interactions, shortening dislocation curvature radius (Figure 11h) and paradoxically lowering CRSS. Thus, the CRSS dependence on precipitation size heterogeneity fundamentally arises from modulation of dislocation path geometry through statistically controlled obstacle encounters.

While the current work focuses on two-dimensional circular precipitates, future studies should extend this approach to investigate the interaction of dislocations with three-dimensional precipitates of arbitrary shapes. Furthermore, incorporating the effects of dislocation climb would present a more comprehensive and physically realistic model.

## 5. Conclusions

In conclusion, this study investigates the combined role of average size and size dispersion of precipitates on the strengthening behavior in nickel-based superalloys. By integrating ML with DDD simulations, our findings suggest that precipitation size dispersion exerts a notable influence on the CRSS, complementing the well-established effect of average size. The ML analysis served as an efficient screening tool, identifying a potential optimal parameter range (average radius of 40–70 nm and size dispersion of 160–300) for further investigation. Subsequent DDD simulations within this range revealed the associated micro-mechanism: a moderate size dispersion promotes the formation of complex dislocation configurations and localized Orowan loops, effectively enhancing dislocation pinning and increasing the CRSS. Our results indicate that while the average size governs the primary strengthening mechanism (shearing vs. Orowan looping), the size dispersion appears to modulate the effectiveness of the Orowan process. An optimal dispersion increases the density of effective obstacles, whereas an excessively broad dispersion reduces it. Therefore, the deliberate control of size distribution, alongside average size, could be a valuable supplementary consideration for the microstructural design of advanced alloys. This integrated approach offers a useful perspective for future research aimed at a more comprehensive understanding of strengthening mechanisms.

## Figures and Tables

**Table 2:** The input and output parameters for machine learning.

Parameter	Input/Output	Descriptions
Average size of precipitates	Input features	Average size of the precipitated phases in DDD simulations.
Distribution of precipitates	Input features	Variance of the radii of the discrete precipitated phases.
Flow stresses	Output features	Average stress after the first drop point of the stress–strain curve.
Yield strength	Output features	The first drop point of the stress–strain curve.

**Figure A1:**
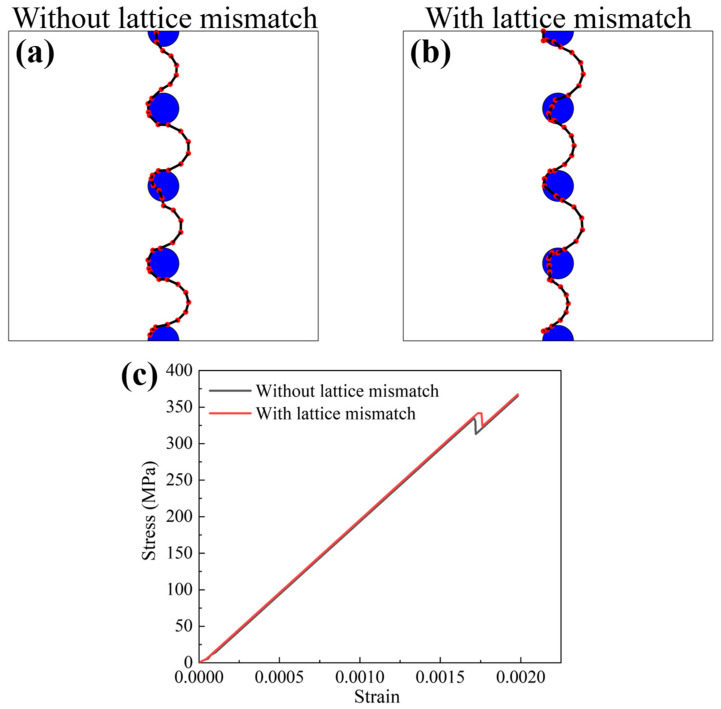
The comparison of dislocation configurations without (**a**) and with (**b**) lattice mismatch. (**c**) Comparison of stress–strain curves with and without lattice mismatch.

## Data Availability

The novel contributions discussed in this study are included within the article. For any additional questions or information, please contact the corresponding author.

## Appendix A. The Influence of Lattice Mismatch

To explore the influence of lattice mismatch dislocation movement in the precipitated phase, we carried out two DDD simulation examples, considering and not considering lattice mismatch, respectively. The size of the precipitated phase is 50 nm (corresponding to the average size of the precipitated phase obtained from the experiment), and it is arranged in a row parallel to the direction of the dislocation line (Figure A1a,b). The stretching direction is [101]. Based on the previous DDD simulation of the interaction between the precipitated phase and dislocations, the stress field induced by the lattice mismatch of the precipitated phase is calculated by the following formula [20]. The eigenstrain is taken as 1%. Similar dislocation configurations (Figure A1a,b) and comparable yield stresses (Figure A1c) indicate that lattice mismatch has little effect on mechanical properties at a precipitation phase size of 50 nm.

The lattice misfit δ quantifies the relative difference in lattice parameters between the precipitate and the matrix, which can be interpreted as a linear eigenstrain.

(A1) $$\varepsilon^{⋆}=\delta I$$

Here, *I* denotes the identity tensor. For an isotropic elastic solid characterized by a shear modulus μ and Poisson’s ratio ν, the resulting stress field generated by the inclusion is expressed using an auxiliary symmetric tensor.

(A2) $$p=2\mu \left(\varepsilon^{⋆}+\frac{\nu }{1-2\nu }tr(\varepsilon^{⋆})I\right)$$

The stress field inside a spherical inclusion of radius a is constant, and it reads

(A3) $$\sigma =2\mu \left[\left(L+\frac{\nu }{1-2\nu }(3L+2M)\right)tr(\varepsilon^{⋆})I+2M\varepsilon^{⋆}\right]-p$$

where $L=\frac{5\nu -1}{15(1-\nu )}$ and $M=\frac{4-5\nu }{15(1-\nu )}$. On the other hand, the stress field of a point x outside the inclusion is

(A4) $$\begin{array}{c} \sigma (x)\begin{array}{cc} = & \frac{a^{3}}{2\left(1-\nu \right)R^{3}}\left[\left(10(1-2\nu )+6\frac{a^{2}}{R^{2}}\right)\frac{p}{15}+\left(2\nu -2\frac{a^{2}}{R^{2}}\right)\frac{\left\{(pr)⊗r+r⊗(pr)\right\}}{R^{2}}\right] \end{array}\\ +\left\{\left(3\frac{a^{2}}{R^{2}}-5\left(1-2\nu \right)\right)\frac{tr\left(p\right)}{15}+\left(1-2\nu -\frac{a^{2}}{R^{2}}\right)\frac{\left(pr\right)\cdot r}{R^{2}}\right\}I\\ +\left\{-\left(5-7\frac{a^{2}}{R^{2}}\right)\frac{(pr)\cdot r}{R^{4}}+\left(1-\frac{a^{2}}{R^{2}}\right)\frac{tr(p)}{R^{2}}\right\}r⊗r \end{array}$$

where r = x − C and **C** is the center of the inclusion and **R** = |**r**|.

## Appendix B. The Dataset Used for Machine Learning

**Table A1:** The full dataset used for training and testing the neural network.

Average Size of Precipitates (nm)	Distribution of Precipitates	Flow Stress (MPa)	Yield Strength (MPa)
30	10	1384.1	1294
50	10	1113.6	1136
70	10	1005.4	990.6
90	10	851.47	849.9
110	10	798.19	778.6
130	10	813.06	816.2
150	10	1035.7	1014
30	20	1263.3	1338
50	20	1068.2	1049
70	20	1308.1	1294
90	20	982.19	960.9
110	20	778.95	775.5
130	20	772.05	768.1
150	20	753.28	750.8
30	40	1370.9	1314
50	40	1138.4	1138
70	40	864.66	863
90	40	844.28	836.7
110	40	893.69	870
130	40	905.07	905.5
150	40	742.04	739.5
30	80	1312.4	1300
50	80	1116.6	1090
70	80	947.07	921
90	80	961.78	965.4
110	80	915.17	886
130	80	784.68	800.4
150	80	701.08	689.5
30	160	1232.8	1182
50	160	1058.5	1206
70	160	1116.4	1062
90	160	875.26	882.4
110	160	900.56	915.7
130	160	775.23	764.8
150	160	809.3	809.5
30	240	1176.7	1096
50	240	1490.8	1424
70	240	1148.8	1233
90	240	1143	1162
110	240	826.28	821.1
130	240	712.78	710
150	240	822	820
30	320	1011.2	958
50	320	1208.2	1252
70	320	1003.6	966.9
90	320	844.94	849.6
110	320	685.5	683.7
130	320	725.23	714.9
150	320	838.54	832.8
